# Origins of Enterovirus Replication Organelles Established by Whole-Cell Electron Microscopy

**DOI:** 10.1128/mBio.00951-19

**Published:** 2019-06-11

**Authors:** Charlotte E. Melia, Christopher J. Peddie, Anja W. M. de Jong, Eric J. Snijder, Lucy M. Collinson, Abraham J. Koster, Hilde M. van der Schaar, Frank J. M. van Kuppeveld, Montserrat Bárcena

**Affiliations:** a Section Electron Microscopy, Department of Cell and Chemical Biology, Leiden University Medical Center, Leiden, The Netherlands; b Electron Microscopy STP, The Francis Crick Institute, London, United Kingdom; c Molecular Virology Laboratory, Department of Medical Microbiology, Center of Infectious Diseases, Leiden University Medical Center, Leiden, The Netherlands; d Virology Division, Faculty of Veterinary Medicine, Department of Infectious Diseases & Immunology, Utrecht University, Utrecht, The Netherlands; University of California, Irvine; Texas A&M University-Texarkana; University of North Carolina at Chapel Hill

**Keywords:** replication organelle biogenesis, coxsackievirus, picornavirus, serial block-face scanning electron microscopy, SBF-SEM, correlative light and electron microscopy, CLEM, volume electron microscopy, membrane structure

## Abstract

Enterovirus genome replication occurs at virus-induced structures derived from cellular membranes and lipids. However, the origin of these replication organelles (ROs) remains uncertain. Ultrastructural evidence of the membrane donor is lacking, suggesting that the sites of its transition into ROs are rare or fleeting. To overcome this challenge, we combined live-cell imaging and serial block-face scanning electron microscopy of whole cells to capture emerging enterovirus ROs. The first foci of fluorescently labeled viral protein correlated with ROs connected to the endoplasmic reticulum (ER) and preceded the appearance of ROs stemming from the *trans*-Golgi network. Whole-cell data sets further revealed striking contact regions between ROs and lipid droplets that may represent a route for lipid shuttling to facilitate RO proliferation and genome replication. Our data provide direct evidence that enteroviruses use ER and then Golgi membranes to initiate RO formation, demonstrating the remarkable flexibility with which enteroviruses usurp cellular organelles.

## Abstract (precis)

Enteroviruses are causative agents of a range of human diseases. The replication of these viruses within cells relies on specialized membranous structures termed replication organelles (ROs) that form during infection but whose origin remains elusive. To capture the emergence of enterovirus ROs, we use correlative light and serial block-face scanning electron microscopy, a powerful method to pinpoint rare events in their whole-cell ultrastructural context. RO biogenesis was found to occur first at ER and then at Golgi membranes. Extensive contacts were found between early ROs and lipid droplets (LDs), which likely serve to provide LD-derived lipids required for replication. Together, these data establish the dual origin of enterovirus ROs and the chronology of their biogenesis at different supporting cellular membranes.

## INTRODUCTION

The production of novel membrane structures is an intriguing and highly conserved feature of positive-sense RNA (+RNA) virus infections. These modified host cell membranes are increasingly referred to as viral replication organelles (ROs), distinct membrane structures that have been suggested to serve as platforms for viral RNA synthesis by coordinating different stages of the viral replicative cycle and/or shielding viral products from innate immune sensors (1–3).

While the formation of ROs during infection is a hallmark of +RNA virus replication, the specific morphologies produced vary by virus. Some viruses (e.g., dengue virus [4] and Zika virus [5]) produce membrane invaginations, or “spherules,” in the membranes of cellular organelles. Other viruses (e.g., hepatitis C virus [6] and severe acute respiratory syndrome [SARS] coronavirus [7]) produce, among other structures, double-membrane vesicles (DMVs) that can be found in isolation or with outer membrane connections to the endoplasmic reticulum (ER), from which they are derived. Identifying the cellular donor organelle for +RNA virus ROs provides important clues about the host factor requirements underlying viral replication. However, determining the donor is problematic when ultrastructural analyses fail to capture direct connections between cellular organelles and ROs. This is the case for the enteroviruses, a large genus of the *Picornavirus* family that includes important human pathogens like poliovirus, coxsackie A and B viruses, several numbered enteroviruses (EVs; e.g., EV-71 and EV-D68), and rhinoviruses.

Enterovirus ROs represent a compositionally and morphologically unique structure in the cells they infect. Their proliferation and utility as replication membranes are dependent on lipids like cholesterol and phosphatidylcholine, which are recruited to ROs via coopted cellular lipid transport mechanisms, and whose levels are sustained by upregulated import, the lipolysis of lipid droplets (LDs), and lipid biosynthesis (8–11). During the earlier stages of infection, enteroviruses produce ROs with a single-membrane tubule (SMT) morphology, which transform into double-membrane vesicles (DMVs) and multilamellar vesicles as infection progresses (12, 13). While enterovirus ROs appear in cytosolic clusters in which their membranes are frequently tightly apposed, both SMTs and DMVs are distinct, isolated structures that do not form a continuous membrane network. These membrane morphologies parallel those found in cells infected with cardioviruses, another genus of *Picornaviridae* (14).

Despite our understanding of enterovirus RO morphology, establishing the sites of their formation has proven challenging, as enterovirus ROs have thus far been observed only as separate compartments that lack direct connections to any cellular organelle. Different studies have diverged in linking RO biogenesis to ER, Golgi, or autophagy membranes (15–18), and their interpretation is complicated by the uncertain correspondence between markers used and ROs. Although the majority of viral RNA (vRNA) synthesis is associated with ROs, it remains possible that the initial sites of genome replication are largely unmodified membranes, ahead of viral protein accumulation and RO biogenesis. In fact, sustained enterovirus RNA synthesis can occur at morphologically unmodified membranes under conditions where RO formation is delayed (19). Moreover, rather than reflecting RO origin, host proteins may be recruited to ROs, independently and differentially, according to infection stage, host cell, or viral species studied. For other +RNA viruses, like SARS coronavirus (7), hepatitis C virus (6), dengue virus (4), and cardiovirus (14), direct connections between host membranes and ROs have been established, providing compelling evidence regarding their origins. While visualizing membrane connections between donor organelle and nascent enterovirus ROs would help clarify whether the ER, Golgi apparatus, or other membranes are utilized for RO formation, the lack of such connections in ultrastructural studies to date suggests that they are rare or transient and thus difficult to capture.

We here utilize correlative light and electron microscopy (CLEM) and serial block-face scanning electron microscopy (SBF-SEM) to overcome this problem and explore the development of ROs at early and advanced stages of enterovirus infection. SBF-SEM is a recently developed technique that facilitates the reconstruction of large volumes (whole cells and tissues) but at the expense of resolution compared to conventional transmission electron microscopy (TEM) (20). First, we explored the resolving power of this technique on enterovirus-infected cells and extracted quantitative information about the abundance and volumes of RO clusters. We next set out to pinpoint the subcellular location of RO biogenesis. For this, we monitored infection until the emergence of the first ROs in live cells, exploiting a split-green fluorescent protein (split-GFP)-tagged coxsackievirus that illuminates the viral 3A protein (21). These emerging 3A foci correlated with nascent ROs in SBF-SEM reconstructions, which were further assessed for any association between cellular organelles and ROs. A close physical association was found between ROs and LDs, whose volumes decreased over the course of infection, suggesting that RO proliferation is supported by the formation of tight LD-RO contacts that facilitate lipid transfer. Importantly, we were able to locate and resolve membrane continuities between putative donor organelles and ROs. Our data provide a timeline that unites apparently disparate observations related to the origins of enterovirus ROs, revealing that RO formation starts at the ER, followed by biogenesis at the *trans*-Golgi network. These findings suggest a remarkable flexibility in virus membrane utilization of different cellular organelles to form morphologically similar structures.

## RESULTS

### Large-volume electron microscopy resolves RO ultrastructure during enterovirus infection.

**FIG 1:**
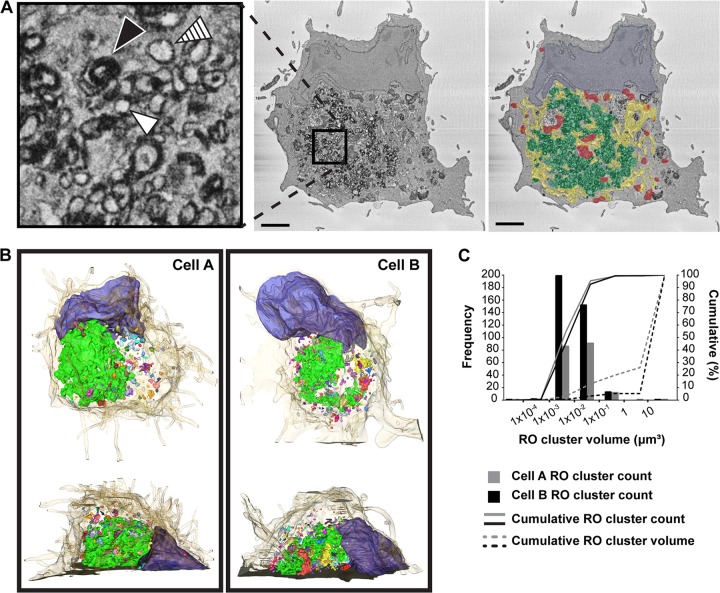
SBF-SEM imaging of CVB3-infected cells resolves ROs in their whole-cell context. Vero E6 cells were infected with CVB3, fixed at 6 h postinfection, and stained and processed for SBF-SEM imaging. (A) Left, a single slice from the SBF-SEM reconstruction of an infected cell. The cytosol is populated by clusters of ROs (example in boxed area), including single-membrane (white arrowhead), double-membrane (hatched arrowhead), and multilamellar (black arrowhead) ROs. Right, cellular organelles, including mitochondria (red), the ER (yellow), and the nucleus (blue), can be resolved alongside ROs (green). (B) Segmentations of two infected cells (A and B) highlight the positions of individual ROs clusters (multicolored) and the nucleus (blue) within the cell volumes (beige, semitransparent). (C) The number of RO clusters and their respective volumes were quantified in the cells presented in panel B, and the histogram of RO cluster sizes revealed a similar binary distribution in each cell, where the majority of RO volume in each cells falls within a single perinuclear cluster, while the majority of all other RO clusters are 2 orders of magnitude smaller. Cells A and B were each collected over 163 sections (8.1-μm depth and total volume of 3,404 μm^3^ per cell). Scale bar = 2 μm.

To assess the resolving power of SBF-SEM on enterovirus-infected cell ultrastructure, Vero E6 cells were infected with coxsackie B virus 3 (CVB3) and fixed at 6 h postinfection (hpi). Our previous studies with electron tomography showed that enterovirus ROs with different morphologies (i.e., single-membrane tubules, double-membrane vesicles, and multilamellar vesicles) are present at this time point. After fixation, cells were prepared and imaged by SBF-SEM along with mock-infected cells for comparison. These data revealed expansive fields of ROs in infected cells, which dominated a large volume of the cell cytoplasm (Fig. 1A; see also Movie S1 in the supplemental material). Although the individual membranes of single- and double-membrane ROs were not resolvable by SBF-SEM under the imaging conditions used, single-membrane ROs (white arrowheads) were distinguishable from double-membrane (hatched arrowheads) or multilamellar (black arrowheads) ROs by the apparent thickness of the stained membrane (Fig. 1A, left). To support this interpretation, SBF-SEM images were compared with higher-resolution TEM images collected from the same type of samples (Fig. S1A and B), confirming that enterovirus ROs were discernible in the SBF-SEM data. Cellular organelles like the ER, lipid droplets, *cis*-Golgi cisternae, and *trans*-Golgi network, could be unambiguously identified in SBF-SEM reconstructions of mock-infected cells through comparisons with TEM images (Fig. S1C). In infected cells, prominent cellular organelles, like the nucleus, ER, and mitochondria, were also identified (highlighted in Fig. 1A, right). While Golgi cisternae were absent throughout these infected cell volumes, Golgi cisternae were clearly visible in all mock cell volumes assessed (*n* = 13). These whole-cell visualizations confirm that Golgi apparatus cisternae disintegrate during enterovirus infection, as was previously inferred from live-cell imaging studies monitoring Golgi markers across infection, and two-dimensional (2D) ultrastructural data at early and late infection time points (12, 17, 19, 22).

10.1128/mBio.00951-19.1 FIG S1 Comparisons of cell ultrastructure in SBF-SEM and TEM images facilitate the identification of ROs and cellular organelles. CVB3-infected or mock-infected Vero E6 cells, chemically fixed for EM and visualized either by SBF-SEM or TEM. (A) Single-membrane, double-membrane, and multilamellar ROs can be distinguished in SBF-SEM data of infected cells by the apparent thickness of the membrane profile. Similar regions imaged by TEM resolve the individual membranes of these structures supporting the interpretation of SBF-SEM images. Single-membrane ROs, white arrowheads; double-membrane ROs, hatched arrowheads; multilamellar ROs, black arrowheads. (B) Line density profiles of the indicated single-, double-, or multimembrane ROs in panel A, as measured along the arrow directions (*x* axis scale in nanometers). (C) Cellular organelles, clearly distinct in TEM images, can also be resolved in SBF-SEM data in uninfected cells. These organelles include Golgi cisternae (white arrowheads, top) and *trans*-Golgi network (hatched arrowheads, top), ER membranes (arrowheads, middle) and lipid droplets (arrowheads, bottom). Scale bars are 2 μm (panel A, top), 500 nm (panel A, boxed regions, and panel C), and 20 nm (panel B). Download FIG S1, TIF file, 2.5 MB. Copyright © 2019 Melia et al. 2019 Melia et al. This content is distributed under the terms of the Creative Commons Attribution 4.0 International license.

10.1128/mBio.00951-19.5 MOVIE S1 Large-volume 3D reconstruction of CVB3-infected cells by SBF-SEM. Consecutive slices through SBF-SEM and segmentation volumes of a Vero E6 cell infected with CVB3 (cell A in Fig. 1) highlighting the distribution of RO clusters (green) in their cellular context (nucleus, blue; cytoplasm, beige) at 6 hpi. The total reconstructed volume was 3,404 μm^3^. Download Movie S1, AVI file, 17.6 MB. Copyright © 2019 Melia et al. 2019 Melia et al. This content is distributed under the terms of the Creative Commons Attribution 4.0 International license.

In order to obtain quantitative insights into the abundance and volumes of enterovirus ROs at this stage of infection, the three-dimensional (3D) distribution of the viral ROs was highlighted by manual segmentation of the RO regions, nucleus, and the cytoplasm in two reconstructed cells, A and B (Fig. 1B). CVB3-induced RO clusters accounted for large parts of the cytoplasmic volume, at 16% of in cell A (104 μm^3^) and 6% in cell B (37 μm^3^). The difference likely reflects a more advanced infection stage in cell A, which is supported by the appearance of extensive ER dilation (Fig. 1A) characteristic of later stages of enterovirus infection (12, 23). The nanometer resolution achievable by SBF-SEM allowed RO clusters in close proximity to be segmented apart to analyze their size distribution (Fig. 1C). For both cells, most ROs were not homogeneously distributed across the cytosol but instead accumulated in a single large cluster (bright green segmented volumes, 98 μm^3^ in cell A and 27 μm^3^ in cell B) proximal to the nucleus. Most of the remaining ROs could be found in relatively small clusters (colored), with more than 90% of these clusters smaller than 0.1 μm^3^ in both cells (Fig. 1C).

### Enterovirus RO biogenesis occurs at distinct ER- or *trans*-Golgi network-derived foci.

Our previous live-cell imaging data demonstrated that the onset of Golgi apparatus disintegration in CVB3 infection is concomitant with the accumulation of viral protein (e.g., 3A) in the Golgi region (19, 21). Intriguingly, the first foci of 3A protein arise in the cell periphery, often ahead of the dramatic 3A accumulation in the Golgi region that expands to dominate the cell cytoplasm as infection progresses (Movie S2, white and black arrowheads, respectively). It is unknown whether both the peripheral and Golgi-proximal 3A foci that emerge during this phase represent developing ROs. One possibility is that the small peripheral 3A foci that form early in infection are replication independent and that these 3A accumulations are merely the result of translation of the incoming vRNA delivered by uncoating of virus particles. To test this, we inhibited vRNA synthesis by treating infected cells with guanidine hydrochloride (GuHCl) and monitored these cells by live-cell imaging. The CVB3 3A was visualized by utilizing a split-GFP system (21). Neither peripheral nor any other 3A-GFP signal emerged under GuHCl inhibition, demonstrating that their formation is not a product of translation of only incoming vRNA but depends upon vRNA replication (Fig. S2).

10.1128/mBio.00951-19.2 FIG S2 The appearance of 3A-GFP foci in the cell periphery requires viral replication. BGM(S1–10) cells were infected or mock-infected with CVB3 3A(S11). The addition of guanidine hydrochloride from 1 hpi, which blocks viral replication, prevented the formation of both peripheral and Golgi-associated 3A-GFP puncta. Download FIG S2, TIF file, 2.4 MB. Copyright © 2019 Melia et al. 2019 Melia et al. This content is distributed under the terms of the Creative Commons Attribution 4.0 International license.

10.1128/mBio.00951-19.6 MOVIE S2 Live-cell imaging of CVB3-infected cells highlights peripheral and Golgi-associated viral 3A foci. BGM(S1–10) cells were transduced with mCherry-GM130 and infected with CVB3 3A(S11) (split-GFP system described in reference 21). As it is typically the case, the first 3A-GFP foci appear in the cell periphery (e.g., white arrowhead, ∼4 hpi), shortly before 3A-GFP signal starts accumulating in the Golgi region (e.g. black arrowhead, ∼4.5 hpi). The disintegration of the Golgi apparatus, apparent by the dispersion and loss of mCherry-GM130 signal, occurred from ∼4.5 hpi through to ∼5.5 hpi. Hatched arrowheads illustrate 3A-GFP foci migration between ∼5 hpi and ∼6 hpi. Scale bar = 10 μm. Download Movie S2, AVI file, 17.1 MB. Copyright © 2019 Melia et al. 2019 Melia et al. This content is distributed under the terms of the Creative Commons Attribution 4.0 International license.

**FIG 2:**
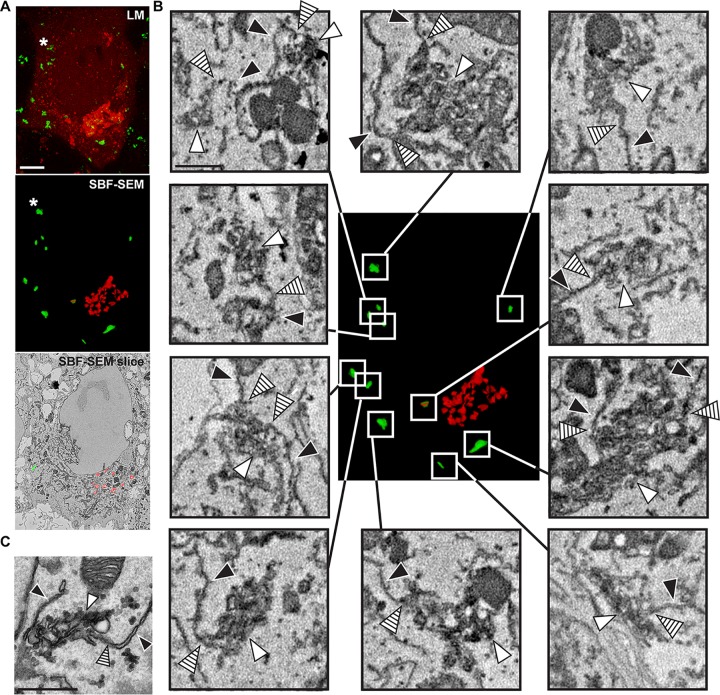
Correlative SBF-SEM and LM highlights the appearance of ROs connected to the ER early in infection. BGM(S1–10) cell transduced with mCherry-GM130, infected with CVB3 3A(S11), and monitored by live-cell imaging until the emergence of the first 3A-GFP puncta. (A) Volume rendering of the final confocal z-stacks prior to fixation and processing for SBF-SEM (mCherry, red; 3A-GFP, green; 4.5 hpi [top]), 3D rendering of the ROs (green) and Golgi cisternae (red) segmented from the corresponding SBF-SEM volume (middle), and a representative SBF-SEM slice (bottom). The intense GM130 signal in confocal data corresponds to recognizable Golgi apparatus cisternae in SBF-SEM data. A good overall correspondence can also be established between 3A-GFP puncta and RO foci (e.g., white arrowheads), despite small discrepancies in their relative position that suggest limited migration of ROs in the delay between LM imaging and fixation (e.g., asterisks). (B) Membrane continuities (hatched arrowheads) between all the RO clusters in the cell (white arrowheads) and ER membranes (black arrowheads) could be observed. (C) Continuities between the ER and ROs could also be found in high-resolution 2D TEM data. Scale bars = 5 μm (A) and 500 nm (B and C).

Next, we investigated whether virus-induced membrane structures could be detected at both peripheral and Golgi-associated 3A foci. To do so, we imaged living cells expressing mCherry-GM130, a *cis*-Golgi marker, and infected them with CVB3 encoding split-GFP-tagged 3A (21). Confocal z-stacks were collected of cells with emerging 3A-GFP signal but at different stages of Golgi disassembly (∼5 hpi), which were then fixed and prepared for SBF-SEM. Acquired SBF-SEM volumes were manually segmented to highlight Golgi cisternae and RO clusters based solely on morphological features, which could then be compared with 3D reconstructions of corresponding confocal data (Fig. 2A, workflow illustrated in Movie S3).

10.1128/mBio.00951-19.7 MOVIE S3 The correlative light and serial block-face scanning electron microscopy workflow. BGM(S1–10) cells were transduced with mCherry-GM130 and infected with CVB3 3A(S11). Confocal z-stacks encompassing the volume of cells of interest were collected, followed by chemical fixation and processing for SBF-SEM. ROs, Golgi cisternae, and lipid droplets were manually segmented from the SBF-SEM volume and rendered in green, red, and yellow, respectively. Comparing both 3D renderings side by side allows establishing the correspondence between LM signal and specific structural motifs. Download Movie S3, MOV file, 12.6 MB. Copyright © 2019 Melia et al. 2019 Melia et al. This content is distributed under the terms of the Creative Commons Attribution 4.0 International license.

A striking correspondence was found between 3A-GFP foci in the confocal data and the location of ROs highlighted by the SBF-SEM segmentation, although in some cases, a shift in the relative positions of 3A-GFP foci and RO clusters was apparent (e.g., hatched arrowheads, Fig. 2A). This may be explained by migration of RO clusters in the delay between confocal z-stack acquisition and fixation (ca. 10 min for CLEM data sets), as peripheral 3A-GFP foci are often dynamic (Movie S2, hatched arrowheads).

The cell presented in Fig. 2 represents an early stage of infection encompassing the emergence of the first peripheral 3A-GFP foci. These foci are largely distal to the Golgi region, where minimal 3A-GFP signal is apparent. The Golgi apparatus appears relatively intact at this stage of infection, as recognizable Golgi cisternae could be detected. All RO clusters at this stage (Fig. 2B, white arrowheads) were observed in close contact with the ER (black arrowheads), with apparent membrane continuities between them (hatched arrowheads). The existence of membrane connections between peripheral RO clusters and ER was confirmed in higher-resolution TEM images of parallel samples (Fig. 2C). These data suggest that 3A foci that emerge in the cell periphery correspond with ROs that are derived from ER membranes.

**FIG 3:**
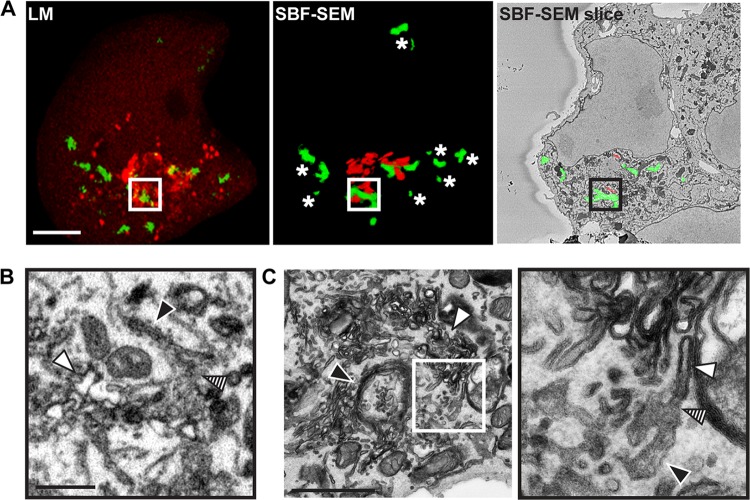
Correlative SBF-SEM and LM exposes RO clusters connected to the Golgi apparatus. BGM(S1–10) cell transduced with mCherry-GM130, infected with CVB3 3A(S11), and monitored by live-cell imaging until the emergence of signal in the Golgi region (∼5 h hpi). (A) Volume rendering of the confocal z-stacks acquired prior to fixation and processing for SBF-SEM (mCherry, red; 3A-GFP, green; 4.5 hpi [left]), 3D rendering of the ROs (green) and Golgi cisternae (red) segmented from the corresponding SBF-SEM volume (middle), and a representative SBF-SEM slice (right). The mCherry-GM130 and 3A-GFP signals correlated well with recognizable Golgi apparatus cisternae and RO clusters, respectively. (B) Assessment of a region (boxed area in panel A) containing Golgi-proximal RO foci reveals a membranous region (hatched arrowhead) bridging ROs (white arrowhead) and the Golgi apparatus (black arrowhead). (C) Overview of a similar region imaged by TEM (left) containing RO clusters (white arrowhead) and the Golgi apparatus (black arrowhead). High-magnification TEM imaging (right; boxed area on the left) reveals membrane continuities (hatched arrowhead) comprising tubules of the *trans*-Golgi network (black arrowhead) and single-membrane RO tubules (white arrowhead). Scale bars = 5 μm (A) and 1 μm (B and C).

Following the emergence of peripheral 3A signal, 3A-GFP accumulates in the Golgi region (Movie S2, white and black arrowheads, respectively). An example of a cell fixed at this stage of infection is shown in Fig. 3A. The 3D confocal model reveals 3A-GFP signal in close proximity to mCherry-GM130 signal, which corresponded to ROs and Golgi cisternae, respectively, in SBF-SEM data (Fig. 3A).

ROs in the Golgi region (Fig. 3B, boxed region in 3A, white arrowhead) were connected to membranous regions that bridged the *trans*-Golgi network (hatched arrowhead) and RO clusters (black arrowhead). Peripheral RO clusters (Fig. 3A, asterisks) were found to be associated with the ER (Fig. S3), demonstrating that ER- and Golgi-associated ROs can coexist in infected cells and suggesting that ER-RO interactions persist at later stages of infection. To examine Golgi-associated ROs at higher resolution, samples prepared for SBF-SEM were imaged in parallel by TEM (Fig. 3C). In addition to Golgi cisternae (Fig. 3C, black arrowhead) and ROs (white arrowheads), TEM images revealed continuities between *trans*-Golgi network tubules and single-membrane ROs (hatched arrowhead). Altogether, these data suggest that early RO biogenesis occurs at seed points distributed across the ER, followed by RO formation at *trans*-Golgi membranes.

10.1128/mBio.00951-19.3 FIG S3 ER-connected and Golgi-derived ROs coexist in CVB3-infected cells. Analysis of the peripheral RO clusters detected in the SBF-SEM reconstruction of the cell is shown in Fig. 3, which was fixed at an early stage of CVB3 infection. The analysis shows that these clusters (boxed regions) correspond to ER-associated ROs that can be formed in addition to the Golgi-derived ROs (see Fig. 3) observed in the same cell. Scale bars = 500 nm. Download FIG S3, TIF file, 2.1 MB. Copyright © 2019 Melia et al. 2019 Melia et al. This content is distributed under the terms of the Creative Commons Attribution 4.0 International license.

### ROs establish extensive contacts with lipid droplets which are depleted during infection.

**FIG 4:**
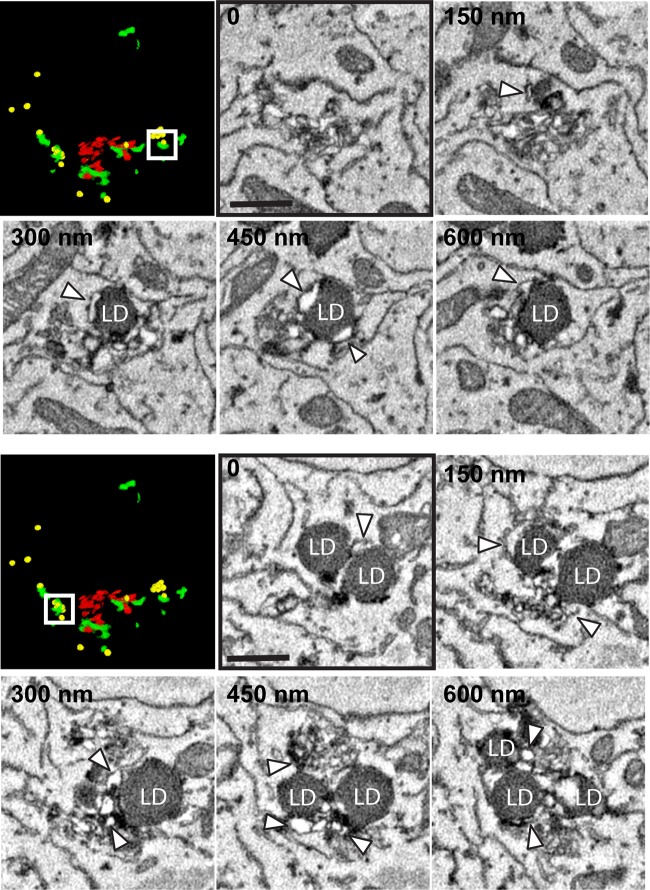
SBF-SEM and TEM data reveal extensive contact between ROs and LDs. Serial SBF-SEM sections from two regions of interest in the cell shown in Fig. 3 (boxed areas in the segmentation models shown; red, Golgi cisternae, green, ROs; yellow, lipid droplets) are presented, highlighting the association between RO tubules (white arrowheads) and lipid droplets (LD). Frame numbers (top left) indicate the relative depth from frame 0 through the SBF-SEM volume. Scale bars = 500 nm.

Beyond the ER- and Golgi-associated RO foci highlighted by the 3A-GFP signal, significant spatial relationships between ROs and other cellular organelles, including endosomes, lysosomes, autophagosomes, and mitochondria, were not clearly apparent in any cells analyzed. However, extensive contact regions could be found between RO clusters and lipid droplets (LDs), which in some cases appeared to be surrounded by ROs (Fig. 4, arrowheads). Interestingly, a qualitative analysis of the number of LDs in SBF-SEM cell reconstructions of cells fixed at late infection stages (*n* = 6) hinted at a possible inverse correlation between the infection stage (assessed by RO abundance) and the LD content. A quantitative analysis of LDs, visualized with Oil Red O stain by light microscopy, confirmed that LDs were depleted across CVB3 infection in our experimental setup and showed a particularly rapid drop early in infection (between 0 and 4 hpi) both in LD counts and the total LD area per cell (Fig. S4), which aligns with previous observations in enterovirus-infected cells (9, 24). Together, our results suggest that the extensive contact regions between ROs and LDs could represent sites for the transfer of critical lipids to ROs, supporting their formation and proliferation.

10.1128/mBio.00951-19.4 FIG S4 Lipid droplets are depleted over the course of infection. Vero E6 cells were infected or mock-infected with CVB3 at an MOI of 100, chemically fixed at the time points indicated, and then stained with Oil red O to visualize lipid droplets. The Oil red O signal as monitored by fluorescence (red) and Hoechst nuclear stain (blue) is shown superimposed onto the differential interference contrast image. (C) The lipid droplet total area (square micrometers) and counts per cell were calculated (25 cells analyzed per time point). A downward trend was observed over the course of infection both in LD volume and count, with significant differences between some time points (**, *P* < 0.05; *, *P* < 0.1, two-tailed *t* test). Error bars represent the standard deviation. Scale bar = 100 μm. Download FIG S4, TIF file, 1.8 MB. Copyright © 2019 Melia et al. 2019 Melia et al. This content is distributed under the terms of the Creative Commons Attribution 4.0 International license.

## DISCUSSION

Establishing the origin of viral ROs reveals important clues about the host cell requirements for their formation. For enteroviruses, existing data suggest that components of the Golgi apparatus, ER, ER exit sites (ERES), autophagy pathway, endolysosomal compartments, or any combination thereof may contribute to the development of ROs (15, 17, 18). Ultrastructural evidence of continuities between cellular organelles and ROs that would shed light on the membrane donor organelle has been lacking, however, suggesting that connections between enterovirus ROs and cellular organelles are rare, or that the association of ROs with their donor organelle is short-lived. To capture these events, we employed live-cell imaging to monitor the emergence of viral 3A protein using a split-GFP system. The resulting 3A-GFP signal was utilized as a correlative marker to highlight potential sites of interest in SBF-SEM cell volumes, which were further assessed for any association between cellular membranes and ROs. These data provide the first direct evidence of host organelle utilization by enteroviruses for RO formation, at both ER and Golgi membranes.

Close examination of SEM volumes revealed connections between peripheral RO clusters and ER membranes as well as membranous regions that bridged perinuclear RO clusters and the *trans*-Golgi network. Higher-resolution TEM images of similar regions confirmed the existence of these membrane continuities. Single-membrane structures, which have been established to be the earliest ROs formed and the precursors of DMVs (12, 13), were predominant both in ER- and Golgi-derived emerging foci, though DMVs could be found in both types. This suggests that the transformation from SMT to DMV is independent of SMT origin.

Peripheral 3A-GFP foci were found to correspond to ER-derived ROs and start to emerge prior to those found in the Golgi region. The proliferation of Golgi-associated 3A-GFP foci was concomitant with Golgi disassembly (21) (Movie S2), and our data suggest that this occurs in conjunction with lipid transfer from the *trans*-Golgi into emerging ROs, a process that could directly contribute to Golgi disassembly. The presence of disperse peripheral RO foci even at late stages of infection (Fig. 1B and C), often in close contact with the ER (data not shown), makes it tempting to speculate that ER-derived ROs are continually produced throughout infection, even after Golgi disassembly. It should be noted that, given the static nature of SBF-SEM data, alternative scenarios cannot be discarded (e.g., RO migration from the large perinuclear cluster).

While the ER- and Golgi-derived membrane modifications described in this work have both been termed ROs based on their characteristic morphology, it remains to be established whether they are functionally equivalent. Given the observation that enterovirus replication can occur at morphologically intact Golgi membranes in a mutant virus under conditions where RO formation is delayed (19), and supported by fluorescence *in situ* hybridization data (17), a strong case can be made that Golgi-derived ROs serve as platforms for viral replication. As for the ER-derived ROs, we show that vRNA synthesis is a requirement for the formation of peripheral 3A-GFP foci and therefore must take place ahead of the development of Golgi-derived ROs. (Fig. S2). At this stage, there is a complete correspondence between peripheral 3A-GPF foci and morphologically typical ER-derived ROs (Fig. 2 and S3). Thus, it appears feasible that both ER- and Golgi-derived ROs are suitable sites for viral RNA synthesis.

Further analyses of SEM volumes did not highlight membrane connections between other cellular structures and ROs but revealed a striking physical association between RO clusters and lipid droplets (LDs) (Fig. 4). While previous light microscopy (LM) data have presented LDs in the vicinity of rhinovirus ROs (24), our high-resolution data revealed that ROs are not only often close to enterovirus LDs, but they also establish extensive contacts with them. In light of a recent study demonstrating the importance of LD-derived lipids for enterovirus replication and RO formation (11), it is tempting to speculate that ROs and LDs can form *bona fide* membrane contact sites (MCS) containing tethers and lipid transfer machinery. These MCSs could underlie an important route for the recruitment of critical lipids for enterovirus RO formation, like fatty acids and cholesterol (8, 25), which may contribute to LD depletion as ROs proliferate over the course of infection (Fig. S4).

Despite differences in the lipid and protein compositions of the ER and *trans*-Golgi network (26), these data demonstrate that apparently morphologically identical enterovirus ROs can be derived from both sites. This indicates that any core cellular components required for enterovirus RO formation are common to both the ER and Golgi apparatus or readily recruited by viral proteins. The enterovirus host factor phosphatidylinositol 4-phosphate (PI4P), which was recently shown to expedite the formation of ROs (19), may represent one example of this. While the beta isoform of phosphatidylinositol 4-kinase, PI4KB, is primarily responsible for PI4P production at the Golgi apparatus, the alpha isoform PI4KA produces PI4P at the ER, with particularly high levels of PI4P present at ERES (27). Together with the observed associations between viral proteins and ERES markers (15, 17), this could nominate PI4P-rich ERES as candidate nucleation points for developing ER-RO foci. However, PI4KA inhibition does not affect the final replication yield during enterovirus infection (28–30), suggesting that ER-derived ROs may confer a small benefit early in infection but are ultimately expendable for replication. Another possibility is that the PI4P utilized for ER-derived RO formation is supplied by PI4KB, recruited by the enterovirus 3A protein (17). While peripheral 3A protein also accumulated early in the replication of a mutant enterovirus, this accumulation was abolished under PI4KB inhibition (19), which could support the notion that ER-derived ROs require PI4KB. In this way, the compositional requirements for RO formation and viral replication would be met by supplementing suitable but diverse donor membranes with recruited host factors.

Altogether, these data demonstrate that direct connections exist between ER and Golgi apparatus membranes and ROs and reveal extensive physical LD-RO contacts that may facilitate the transfer of lipids from LDs to ROs. Correlative live-cell and SBF-SEM imaging indicates that RO formation occurs at ER and then Golgi membranes with some chronological separation, implying that the core cellular components required for enterovirus replication and RO formation are common to both organelles or readily recruited by viral proteins. Flexible recruitment of membranes for replication would confer a remarkable level of adaptability to different conditions, providing +RNA viruses with an important evolutionary advantage. This work extends the growing body of evidence suggesting that +RNA viruses are not constrained to utilizing membranes from a single cellular source for their replication, with important implications for the development of antivirals targeting viral host factors.

## MATERIALS AND METHODS

### Cell lines and reagents.

Vero E6 or BGM cells were cultured using Dulbecco’s modified Eagle’s medium (Gibco) supplemented with 10% fetal calf serum, penicillin, and streptomycin, and maintained at 37°C in 5% CO_2_. BGM(S1–10) cells, which express GFP(S1–10) and the puromycin resistance gene (Pac), have been described in reference 21. BGM(S1–10) cell culture medium was supplemented with an additional 30 μg/ml puromycin as a selection agent.

### Viruses and infection.

CVB3 3A(S11aa2), which encodes a 3A with the S11 region of GFP inserted after amino acid position 2, has been described in (21). CVB3 wild type (wt; strain Nancy) was generated as described in reference 19. For infection, cells were inoculated for 1 h with CVB3 wt or CVB3 3A(S11) at MOIs of 50 and 5, respectively, after which the inoculum was removed and fresh medium was added. At specified time points after infection, cells were imaged by light microscopy and/or prepared for electron microscopy by chemical fixation.

### Live-cell imaging.

BGM(S1–10) cells were grown to ∼35% confluence in glass-bottom 4-chamber 35-mm dishes (CELLview) and transduced with murine leukemia virus (MLV) mCherry-GM130 particles (described in reference 21). Transduced cells were infected with CVB3 3A(S11) 18 to 24 h later. Cells were washed with FluoroBrite medium (Thermo Fisher Scientific) supplemented with 8% fetal calf serum (FCS) and 25 mM HEPES just prior to imaging. LM data were collected using a Leica SP5 confocal microscope equipped with a HyD detector and a 63× (1.4 numerical aperture [NA]) oil immersion objective, with the confocal pinhole adjusted to 1 airy unit for GFP emission (95.56-μm pinhole). For time-series imaging, positions of interest (*xyz*) were marked and imaged sequentially at 5-min intervals. For the duration of all imaging, cells were maintained at 37°C and 5% CO_2_ in a live-cell chamber. For correlative light and electron microscopy, cells were grown in glass-bottom MatTek dishes with an etched alphanumeric coordinate system (MatTek Corporation) to facilitate the relocation of the regions identified during light microscopy. Cells were monitored until the point of interest, at which time a confocal z-stack was acquired, after which samples were immediately fixed for electron microscopy. To minimize the time between the first image acquisition of each z-stack and fixation (ca. 10 min total), one field of view was imaged per sample.

### Lipid droplet visualization and quantification.

Vero E6 cells grown on coverslips were infected with CVB3 wt (MOI 100) and fixed with 3% paraformaldehyde for 1 h at different infection times. Mock-infected cells were also fixed as a control. After washing with PBS, the coverslips were incubated for 1 h at room temperature with a freshly prepared mixture of Oil Red O (Sigma) (0.5% [wt/vol] in isopropanol) and double-distilled water (6:10) to stain LDs, washed with PBS, and incubated with the nuclear stain Hoechst. The samples were then imaged in a Leica DM6 wide-field microscope with a 40× oil immersion objective using both differential interference contrast (DIC) and fluorescence imaging modes. LDs appeared as darkly stained circular profiles in the DIC images that were absent in unstained samples and could be also detected in fluorescent images using a Texas Red excitation filter (540 to 580 nm) (31). A total of 25 cells per condition were randomly selected from the DIC images, and the LDs contained in those cells were counted and manually segmented from the fluorescent images using Amira 6.0.1 (Thermo Fisher), which was further used to compute the LD area per cell.

### Electron microscopy.

**(i) Preparation of chemically fixed samples.** Sample fixation, staining, and embedding procedures were adapted from reference 32. Cells were fixed with 2.5% glutaraldehyde and 4% formaldehyde in 0.15 M cacodylate buffer for 60 min at room temperature. Initial postfixation was carried out using a solution of 2% osmium tetroxide and 1.5% potassium ferricyanide in 0.15 M cacodylate for 60 min on ice. Samples were then treated with 1% aqueous thiocarbohydrazide for 20 min, followed by 2% aqueous osmium tetroxide for 30 min, and were finally incubated overnight at 4°C in 1% aqueous uranyl acetate. Samples were then stained using Walton’s lead aspartate at 60°C for 30 min ahead of stepwise dehydration in ethanol. Samples were infiltrated with resin initially using a 50/50 mixture of propylene oxide and Durcupan ACM resin (Ladd Research) for 60 min. Where MatTek dishes were used, coverslips were excised from the surrounding culture dish using a razor blade and removed to a container resistant to propylene oxide ahead of this step. Samples were then infiltrated with fresh undiluted Durcupan for 90 min before covering regions of interest with inverted resin-filled BEEM capsules (Ted Pella) and polymerization for 48 h at 60°C. For TEM, sections of 70 nm were cut from the sample block for imaging and poststained with uranyl acetate and lead citrate. For SEM, sample blocks were prepared as described in reference 33. Briefly, small portions of the cell monolayers were mounted on pins using conductive epoxy resin (CircuitWorks CW2400), trimmed to form a pillar approximately 400 by 400 by 150 μm, ensuring that regions of interest were retained in the block-face for correlative samples, and coated with a 2-nm layer of platinum ahead of imaging.

**(ii) Electron microscopy imaging.** TEM images were collected in an FEI Tecnai 12 BioTWIN microscope using an Eagle 4K slow-scan charge-coupled-device (CCD) camera (FEI) or an FEI Twin at 120 kV with OneView 4K high-frame-rate camera (Gatan), both in binning mode 2. Serial block-face scanning electron microscopy (SBF-SEM) data were collected using a 3View 2XP system (Gatan, Pleasanton, CA) attached to a Sigma VP SEM (Zeiss, Cambridge, United Kingdom). Back-scattered electron images were acquired using the 3VBSE detector. The SEM was operated at a chamber pressure of 5 Pa, with high-current mode inactive. Variable pressure imaging conditions, which reduce the final resolution, aided in the suppression of charging resulting from the large regions of bare resin surrounding single cells. For CLEM data, the 30-μm aperture was used with an accelerating voltage of 2 kV, a dwell time of 3 μs (10-nm reported pixel size; horizontal frame width, 81.9 μm), and 40-nm slice thickness. Two collection setups were used for samples fixed at 6 hpi, using either the 30- or 20-μm aperture and an accelerating voltage of 2 or 3 kV, respectively. The imaging dwell time was 3 μs (5-nm reported pixel size; horizontal frame width, 20.5 μm) or 10 μs (6-nm reported pixel size; horizontal frame width, 24.6 μm), with a slice thickness of 50 or 30 nm.

**(iii) SBF-SEM image processing and segmentation.** SBF-SEM image stacks were acquired as dm3 files and converted to 32-bit tagged image files (TIFs) for initial batch processing. Images were Gaussian filtered (1 pixel), followed by two rounds of unsharp mask and gray-level normalization (Adobe Photoshop). Image stacks were then converted to 8-bit TIFs and aligned using the virtual align slices plugin (Fiji). Segmentation of features within SBF-SEM data was carried out in a semiautomatic threshold-based way for the cell cytoplasm and manually for the viral ROs, nuclei and LDs using either Segmentation Editor (Fiji) or Amira 6.0.1 (Thermo Fisher), which was then also employed to extract volume information on the different RO clusters.
