# Supplementary figures and images for: Origins of Enterovirus Replication Organelles Established by Whole-Cell Electron Microscopy
Source: mBio. 2019 Jun 11;10(3):e00951-19. doi: 10.1128/mBio.00951-19 (PMC6561026; doi:10.1128/mBio.00951-19)

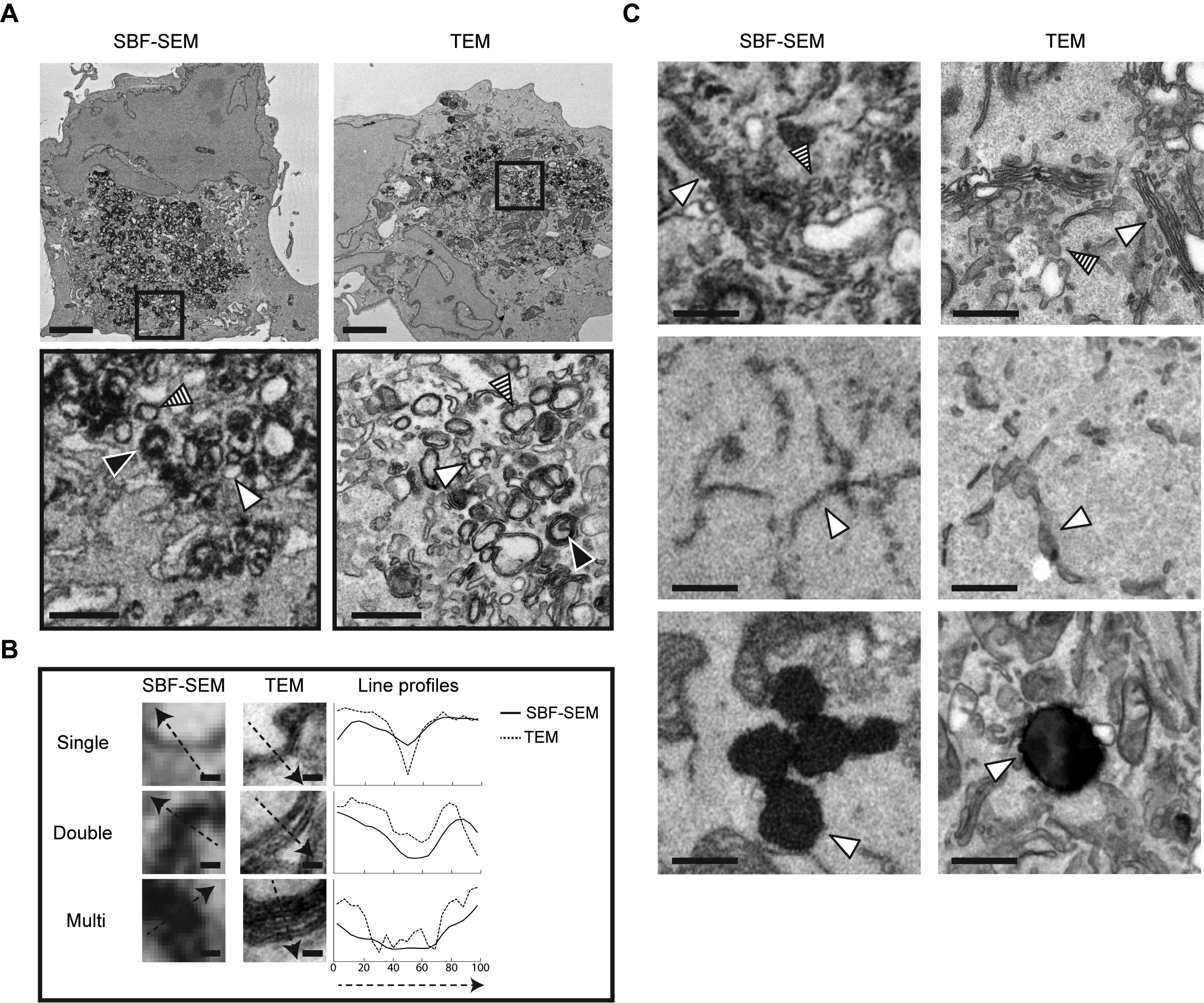

Supplement: FIG S1 [file mBio.00951-19-sf001.tif]

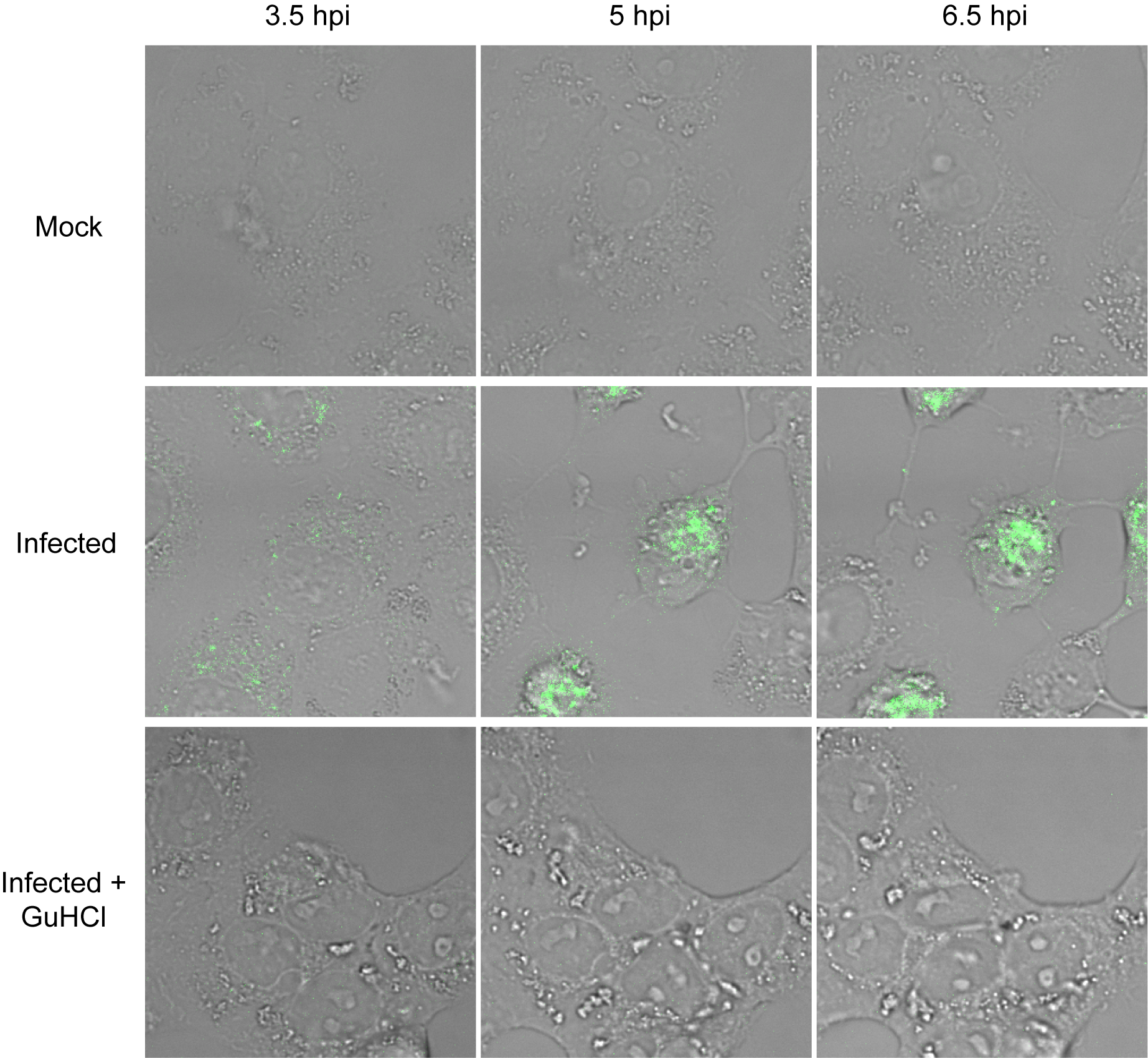

Supplement: FIG S2 [file mBio.00951-19-sf002.tif]

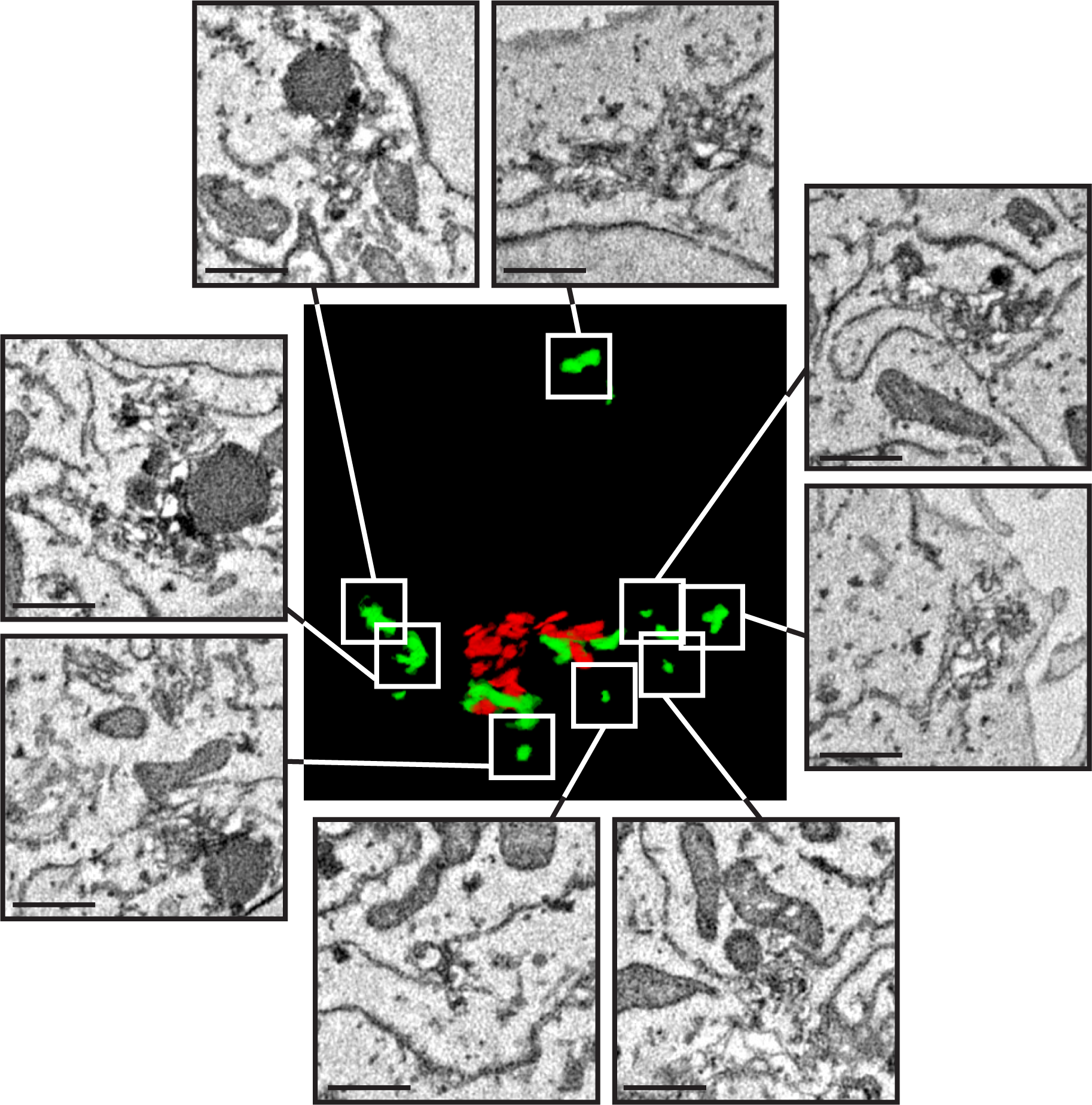

Supplement: FIG S3 [file mBio.00951-19-sf003.tif]

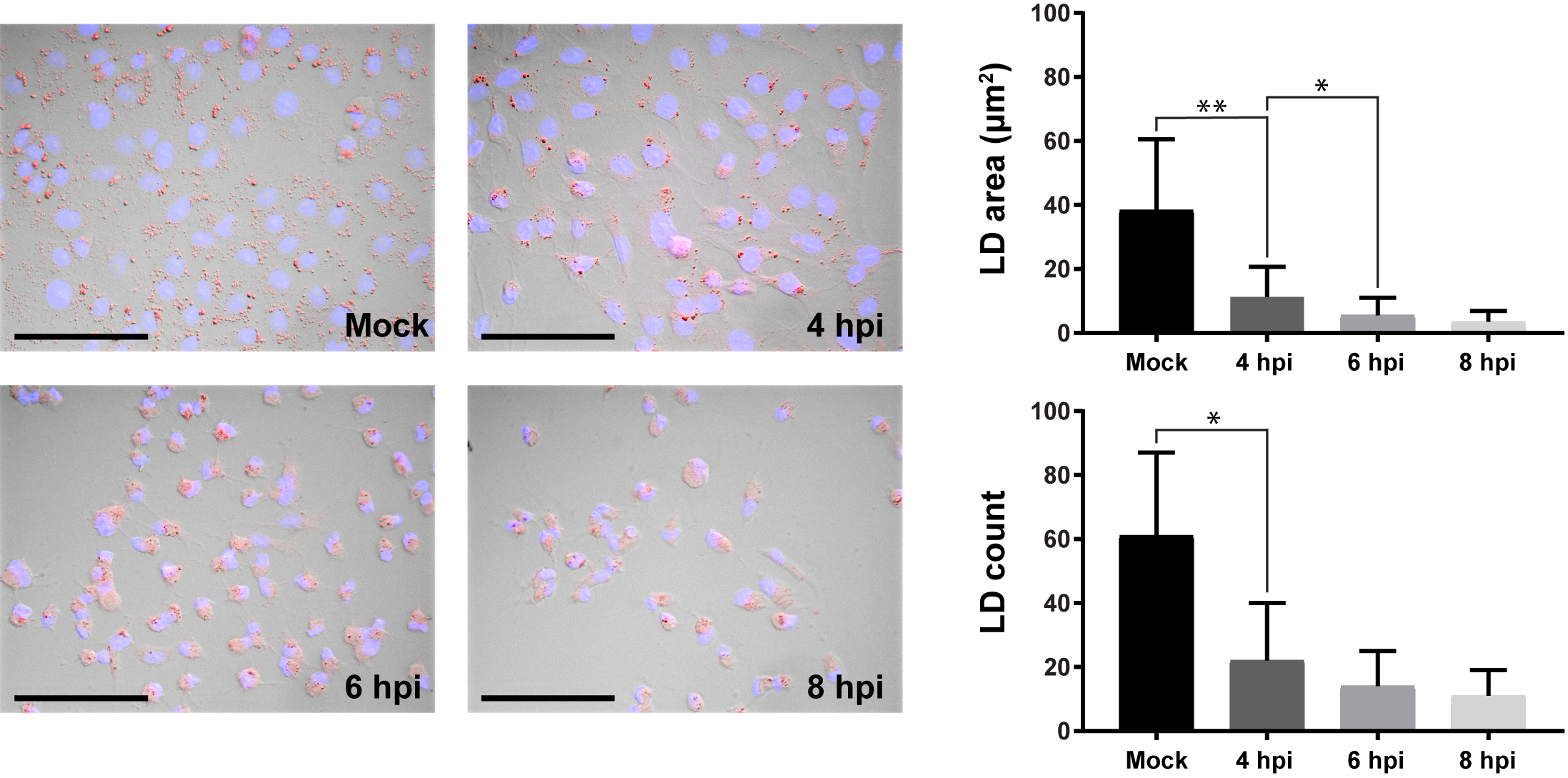

Supplement: FIG S4 [file mBio.00951-19-sf004.tif]
